# Adipose-Tissue Macrophage Diversity and Functions

**DOI:** 10.3390/ijms27041759

**Published:** 2026-02-12

**Authors:** Sacha Grenet, Stoyan Ivanov, Giulia Chinetti

**Affiliations:** 1 Faculté de Médecine, Université Côte d’Azur, CNRS, LP2M, 28, Avenue de Valombrose, 06107 Nice, Cedex 2, France; sacha.grenet@univ-cotedazur.fr (S.G.); stoyan.ivanov@univ-cotedazur.fr (S.I.); 2 Centre Hospitalier Universitaire de Nice, 30, Voie Romaine, 06107 Nice, France

**Keywords:** inflammation, obesity, immune cells, macrophages

## Abstract

Macrophages are the predominant immune cell type found in adipose tissue (AT). They play a critical role in tissue homeostasis and participate in metabolic regulation. In white adipose tissue (WAT), studies show that multiple macrophage subsets co-exist in the very same microenvironment. Yet these cells express selective membrane markers, allowing for identifying several well-distinguished populations. In the present review we discuss the diversity and functions of white-adipose-tissue macrophages. We summarize current knowledge regarding the intratissue distribution of macrophage populations and their specific association with stromal cells and discuss the mechanisms governing adipose-tissue macrophage generation and survival. We emphasize the central role of adipose-tissue macrophages in handling local lipid levels. A particular focus is placed on the recently described population of lipid-associated macrophages (LAMs), particularly abundant in adipose tissue during obesity development.

## 1. Introduction

### 1.1. Adipose-Tissue Macrophages

Adipose tissue contains multiple innate and adaptive immune cell populations. Macrophages are the numerically predominant immune cell type in adipose tissue at steady state. Several macrophage subsets have been described in thermogenic (brown adipose tissue (BAT)) and non-thermogenic (white adipose tissue (WAT)) fat depots [1,2,3].

White adipose tissue contains both resident adipose-tissue macrophages (ATM) as well as newly recruited monocytes that are the major cells contributing to increased ATM number during obesity both in mice and humans [4,5]. While multiple ATM subsets exist in mouse and human adipose tissues, their respective roles and functional differences are still not fully understood. The diversity and functions of brown-adipose-tissue macrophages have been extensively reviewed recently [6] and in the current manuscript we will focus our attention on white-adipose-tissue macrophages.

### 1.2. Adipose-Tissue Macrophage Development, Maintenance and Functions

Most tissue-resident macrophages depend on the growth factor CSF1 (colony-stimulating factor) for their generation survival. CSF1 binds to its receptor CSF1R (CD115), whose expression in rodents is restricted to the myeloid cell progenitors, monocytes and macrophages [7]. CSF1 and CSF1R deficiency are associated with macrophage absence in multiple tissues in mice and rats. In young CSF1R^−/−^ rats, white adipose tissue is completely lacking while interscapular BAT is still detected [8]. Similar data were also obtained in mice, in which CSF1R deletion specifically in myeloid cells is associated with decreased adipose-tissue size [9]. These data indicated a central role of CSF1R-dependent monocytes and macrophages in WAT physiology. TRIB1-deficient mice, which display a partial reduction in F4/80^+^CD206^+^ ATMs, are characterized by decreased WAT size [10]. These mice have increased adipose-tissue lipolysis culminating in elevated systemic fatty acid and glycerol concentrations [10]. Importantly, the authors found that the expression of genes involved in lipogenesis was not affected in TRIB1-deficient animals [10]. Thus, WAT macrophages control adipocyte lipolysis and contribute to tissue homeostasis and lipid mobilization.

In mice, ATMs highly express Platelet-Derived Growth Factor cc (PDGFcc) [9]. PDGFcc deletion in CSF1R-expressing cells triggered decreased WAT, but not BAT, size [9]. Adipocyte size was significantly decreased in PDGFcc–myeloid cell-deficient mice, suggesting a central role of monocytes and macrophages in lipid storage [9]. The authors demonstrated that PDGFcc is a critical regulator of adipogenesis. Indeed, in the absence of PDGFcc, excess lipids that could not be stored in WAT are directed to other tissues [9]. Thus, WAT macrophages are involved in both lipid storage and their mobilization. Yet one could wonder whether those functions are fulfilled by the very same macrophage subset or dedicated macrophage populations are controlling separate metabolic pathways in adipocytes.

**Figure 1:**
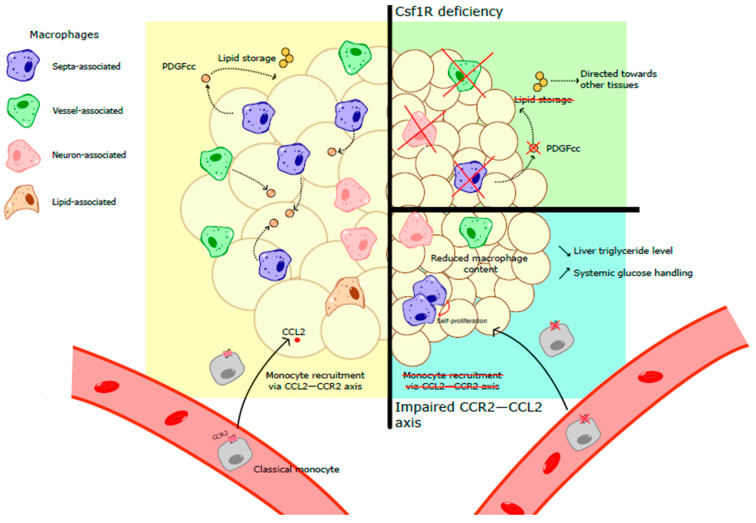
Macrophage-dependent regulation of ATs and lipid metabolism depends on both CSF1–CSF1R and CCL2–CCR2 axes. CSF1–CSF1R axis is crucial for the development and survival of WAT macrophages. These cells play a critical role in lipid storage via factors such as PDGFcc. In obesity, monocyte recruitment via the CCL2–CCR2 axis and local proliferation increase macrophage number in AT, which contributes to both local inflammation and systemic metabolism alteration. Cross-out elements are absent in the corresponding condition. Black arrows represent cell migration, red arrow is self-proliferation, dashed arrows are cytokines secretion or signaling.

Obesity is an important risk factor for the development of cardio-metabolic diseases such as atherosclerosis and type 2 diabetes. The prevalence of obesity in modern societies has reached pandemic proportions and represents a critical burden for healthcare systems [11]. Obesity is characterized by a low-grade inflammation and subsequent macrophage accumulation in WAT [4,5,12]. The increased number of ATMs could be a consequence of two highly complementary mechanisms: monocyte recruitment from the blood circulation; proliferation of tissue-resident macrophages. Monocyte recruitment into inflamed tissues is mediated by the chemokine receptor CCR2 [13]. CCR2 binds to several chemokines, the most characterized being its interaction with CCL2 (MCP1). CCL2 is mainly produced by monocytes/macrophages and endothelial cells. In obese mice, CCL2 levels in white adipose tissue progressively increased [14,15]. This observation suggests that monocyte recruitment, rather than self-proliferation, contributes to the increased macrophage number in obese mice. However, another report demonstrated that the CCL2/CCR2 axis mediates macrophage proliferation in adipose tissue during obesity development [16]. Mice overexpressing CCL2 in adipose tissue are characterized by increased macrophage content [15]. These animals present increased plasma fatty acid concentration and hepatic triglycerides [15]. In contrast, CCL2-deficient mice display reduced fatty acid and triglyceride levels [15]. Similarly, CCR2-deficient mice fed on a high-fat diet present reduced ATM content [17]. This is associated with lower liver triglyceride levels and improved systemic glucose handling [17]. Importantly, in CCR2^−/−^ animals the reduced monocyte recruitment in peripheral tissues is at least partially compensated by increased proliferation of tissue-resident macrophages [18]. Thus, macrophage subset diversity is affected in CCR2-deficient mice and macrophages displaying markers of prolonged tissue residency are enriched in those mice (Figure 1). Of note, it currently remains to be established whether monocyte-derived and embryonically seeded adipose macrophages compete for local CSF1. Whether CSF1R affinity differs depending on macrophage origin is a critical question that needs to be elucidated. Furthermore, the identity of CSF1-producing cells in WAT, both at steady state and during obesity, remains to be established.

### 1.3. Diversity of Adipose-Tissue Macrophages

**Figure 2:**
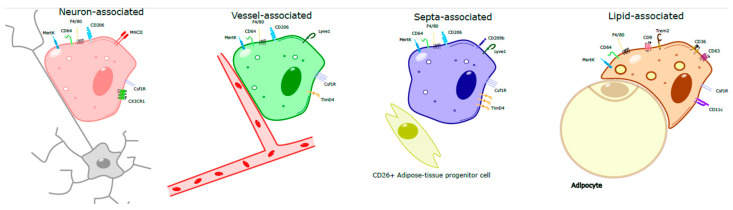
Phenotypic diversity of adipose-tissue macrophage subsets. Phenotypically distinct macrophage subtypes are found in ATs, such as vessel-associated (LYVE1+), neuron-associated (CX3CR1^+^ MHC-II^high^), septa-associated (CD209b^+^), and lipid-associated macrophages (LAMs; Trem2^+^, CD9^+^). These populations co-exist in different niches and have specialized functions.

Several macrophage populations expressing distinct phenotypic markers co-exist in healthy and inflamed adipose tissue (Figure 2) [19,20,21]. In mice and humans, obesity development is associated with marked modulation of ATM phenotype towards a pro-inflammatory phenotype [22,23]. In lean mice and humans, the predominant macrophage population is characterized by the concomitant expression of CD11b, F4/80 and CD206 [24]. The proportion of ATMs in obese patients represents approximately 40% of the adipose-tissue immune cells, while it reaches only 10% in lean people [4].

While several ATM subsets have been described based on the expression of peculiar markers or spatial location, allowing for identification of specific cell functions related to pathophysiological states (lean vs obese, for example), it cannot be excluded that mixed ATM phenotypes can exist depending on AT microenvironment that vary during obesity progression, thus rendering their identification and characterization even more complex.

In lean subjects, ATMs expressing CD14, CD68, CD163, CD204, and CD206 mainly exert homeostatic and regulatory functions, thus having a potential protective role. Indeed, they control lipids and free fatty acids released by adipocytes, thus limiting lipotoxicity, and interact with adipocytes, contributing to metabolic equilibrium maintenance. ATMs also participate in the phagocytosis of dead adipocytes, thus reducing adipose-tissue stress and producing anti-inflammatory cytokines (IL-10) [25].

During obesity development, increased numbers of CD11c^+^ (pro-inflammatory) macrophages were documented in adipose tissue and these cells were shown to contribute to tissue inflammation and insulin resistance. These cells secrete pro-inflammatory factors such as TNFα, IL-1β, IL-6 and nitric oxide [26]. Depletion of pro-inflammatory macrophages is associated with improved systemic glucose metabolism [23,27,28]. Yet one could wonder if the effect is fully mediated by adipose-tissue macrophages since the depletion strategies applied likely affected macrophages and other immune cells in multiple organs. Thus, there is still an urgent and unmet need to develop and validate tools allowing for specifically targeting adipose-tissue macrophages and exploring their role in the maintenance of tissue homeostasis and during metabolic diseases.

Advances in spatial transcriptomics and resolved single-cell analyses have recently provided novel insights into the spatial distribution of ATM subsets within specific niches, thus shaping macrophage phenotype and functions [29]. Indeed, among immune cells, pro-inflammatory macrophages as well as B cells and mast cells were commonly identified in clusters in human adipose tissue, mainly located in crown-like structures (CLS), while anti-inflammatory macrophages, together with NK cells, were dispersed across the tissue [30].

Further analysis suggested a connection between fibro-adipogenic precursors (FAPs) and anti-inflammatory macrophages in human subcutaneous WAT [31]. Moreover, a direct crosstalk between macrophages and FAPs involving chemerin and IL-16 signaling was predicted [31].

The majority of ATMs are located nearby to and closely interact with blood vessels [3]. These cells highly express the membrane marker LYVE1 and the mannose receptor CD206 and efficiently sample blood-borne material [3,32,33]. Interestingly, LYVE1^+^ macrophages display a higher expression of CSF1R in comparison to LYVE1^−^ macrophages [33]. Among LYVE1^+^ adipose-tissue macrophages, a cluster of cells express CD209b [34]. These macrophages are located in contact with adipose-tissue septa and regulate the fate of adipocyte progenitors [34]. The depletion of CD209b^+^ macrophages was associated with increased adipose-tissue beiging and the induction of a thermogenic program, further providing evidence that ATM subsets have highly dedicated and complementary functions.

A second macrophage subset expressing CX3CR1 and MHC-II was also found in adipose tissue [32]. These cells reside near neurons and presumably control local catecholamine content and adipose-tissue lipolysis [35]. This macrophage subset expresses the catecholamine transporter Slc6a2 allowing to internalize norepinephrine and the enzyme monoamine oxidase (Maoa) involved in its degradation [35]. While previous work suggested that ATMs are involved in norepinephrine production [36], more recent data demonstrated that these cells do not have the intracellular enzymes involved in norepinephrine synthesis [37]. A population of CD169^+^ neuron-associated macrophages was recently identified [38]. The number of these cells decreased with age and their depletion was associated with diminished adipose-tissue lipolysis [38]. Whether neuron-associated ATMs are a heterogeneous cell population remains to be fully determined.

TRIB1 mutations have been associated with lipid metabolism modulations in humans [39,40]. As discussed above, TRIB1 plays a major role in ATMs [10] and therefore one could wonder whether dysregulation in systemic lipid metabolism could be due to macrophages.

Among ATMs, metabolically activated macrophages (MMMe) have been identified both in obese mice and humans in environments rich in palmitate, insulin and glucose [41]. These cells express ATP-binding cassette type 1 (ABCA1), perilipin 2 (PLIN2) and CD36, as well as IL-1β. Moreover, they are active in the clearance of dead adipocytes via lysosomal exocytosis, thus inhibiting ectopic fat accumulation. Their pro-inflammatory as well as adipocyte-clearing properties depend on the NADPH oxidase 2 (NOX2) pathways. Thus, MMe macrophages perform detrimental and beneficial functions contributing to metabolic phenotypes during obesity progression [41].

Perhaps the most studied macrophage subset in adipose tissue are the lipid-associated macrophages (LAMs), which are related to but differ from MMes. LAMs play a central role in the maintenance of tissue homeostasis and in handling lipid metabolism [42]. Indeed, LAMs uptake and metabolize excessive lipids to prevent their accumulation, which could trigger metabolic disorders. Steady-state LAMs in adipose tissue are scarce and their numbers increase during obesity development [19]. LAMs express a specific set of markers allowing to distinguish them from the other macrophage subsets discussed above. Among these markers are Trem2, CD9, Spp1, CD36, CD63, Lpl and Fabp5 [19,43]. They also possess a transcriptomic signature associated with lipid metabolism. Using genetic models, it was demonstrated that LAMs prevent obesity development in mice [19,44]. Indeed, Trem2-deficient mice develop larger adipocytes and are characterized by increased adipocyte death upon high fat diet (HFD) feeding [44]. This was associated with augmented adipose-tissue inflammation [44]. Moreover, LAMs highly and selectively express TM4SF19, a transmembrane 4 L six family member 19 lysosomal protein [45]. Mice lacking TM4SF19 specifically in macrophages displayed reduced adipose-tissue macrophage content, associated with improved metabolic parameters [45].

Adipose LAMs are a heterogenous population that could arise from both tissue-resident macrophages and from blood monocytes [43]. Yet, whether adipose-tissue LAM ontogeny could modulate their functions and confer a specificity to handle selective lipid species remains to be established. A subset of anti-inflammatory ATMs derived from recently differentiated macrophages and in the process of acquiring the LAM phenotype, consistent with a LAM precursor phenotype (pre-LAM), was identified in early obesity, spatially associated with CLS. Spatial analyses revealed the colocalization of transcripts among monocytes, pre-LAMs, and LAMs, including *Apoe*, *Lrp1*, *Lpl*, and *App* [46]. While pre-LAMs were abundant in early obesity, LAMs were increased in chronic obesity.

The expression of LAM markers (including TREM2, CD9, CD68, and GPNMB) was found to be higher in CD11b^+^ cells in subcutaneous adipose tissue (scAT) compared to visceral adipose tissue (VAT), the latter expressing higher levels of LYVE1, TIMD4 and MRC1 in obese patients [47]. Of note, CD248, a marker associated with insulin resistance, was found to be highly expressed in scAT-infiltrated CD11b^+^ cells compared to VAT. Indeed, the pathological significance of these differences in obesity will require further studies [4].

Moreover, deep adipose tissue (DAT) is more strongly associated with metabolic abnormalities, particularly obesity, than scAT, which presents a higher proportion of anti-inflammatory macrophages, whereas DAT is characterized by oxidative-stress-associated macrophages (Mox), thus showing a pro-inflammatory profile [48]. Based on the expression of CD9 (member of the tetraspanin family), two different ATM populations have been identified in human obese visceral WAT [21]. CD9^+^ ATMs, which are located prominently in CLS, express higher levels of CD16 and CD206 and have higher intracellular lipids compared to CD9^−^ ATMs. The number of CD9^+^ ATMs is positively correlated with BMI (body mass index), thus suggesting a pathophysiological role of this ATM subset.

Indeed, a subset of ATM involved in iron handling, the MFe^hi^ macrophages, have also been reported in obese mice and humans [49]. Located in the intracellular space of adipose tissue, these macrophages express CD163, ferritin and ferroportin and display higher iron content and the ability to recycle iron. Thus, they protect adipose tissue from iron overload. While LAM appearance is associated with obesity initiation and development, a clear consensus on the mechanisms accounting for LAM generation is not yet established. Whether LAM development reflects on a specific cell-intrinsic program allowing to generate cells instructed to handle large lipid amounts or, alternatively, the appearance of LAMs is a direct consequence of a lipid overload in the microenvironment remains to be defined.

## 2. Conclusions and Future Directions

While mouse and human adipose-tissue macrophages share multiple similarities, reflecting the phenotypical plasticity of these cells, several key differences were also documented. Notably, while in mouse adipose-tissue macrophage subsets could be identified based on their expression of LYVE1 and MHC-II, in humans all adipose-tissue macrophages express HLA-DR [32]. Thus, the phenotype of neuron-associated adipose-tissue macrophages in humans remains to be established. The significant overlap in LAM characteristics between mice and humans demonstrates that preclinical mouse models are valuable tools for investigating LAM functions in human diseases. Yet, due to limited tissue accessibility, data analyzing human macrophage functions, in particular in thermogenic adipose tissues, are scarce. The advent of novel technologies in the past decade allowed for illustrating ATM diversity in humans and their intratissue distribution. However, whether the functions of macrophage subsets identified in mice are conserved in humans require further research.

Because LAMs play a central role in lipid metabolism, better defining their functions could lead to the development of novel therapeutic strategies. To identify molecular pathways and allow for amplifying or decreasing LAM abundance in adipose tissue is an exciting area of research. Therefore, a better comprehension of the mechanisms allowing for generating specific ATM subsets, at steady state and upon inflammation, could provide the opportunity to tailor novel strategies to modulate adipocyte numbers and content, local inflammatory responses and finally to prevent metabolic diseases.

## Data Availability

No new data were created or analyzed in this study. Data sharing is not applicable to this article.
